# Transferring healthcare risk prediction models between Medicaid populations: a transfer learning evaluation

**DOI:** 10.1038/s44401-026-00097-w

**Published:** 2026-06-19

**Authors:** Sanjay Basu, Sadiq Y. Patel, Parth Sheth, Bhairavi Muralidharan, Namrata Elamaran, Aakriti Kinra, Rajaie Batniji

**Affiliations:** 1 Waymark, San Francisco, CA USA; 2 University of California San Francisco, San Francisco, CA USA; 3 University of Pennsylvania, Philadelphia, PA USA

**Keywords:** Health care, Mathematics and computing, Medical research

## Abstract

Cross-state deployment of Medicaid risk prediction models is challenged by demographic, policy, and care-delivery differences that create domain shift. We evaluated transfer learning methods for predicting acute care utilisation between Washington (source; *n* = 20,744) and Virginia (target; *n* = 28,901) Medicaid populations enrolled in high-risk care management, where outcome prevalence differed markedly (9.4% vs 25.6%). Nine approaches were compared: source-only and target-only logistic regression, prototypical networks, domain-adversarial neural networks, causal transfer learning, TabTransformer, Enhanced MAML, a meta-ensemble, and a simple average ensemble. On the Virginia hold-out set, the meta-ensemble achieved the highest discrimination (AUC 0.728, 95% CI 0.691–0.764) and best calibration (Brier 0.193, 95% CI 0.180–0.207). Source-only transfer performed similarly (AUC 0.725), with no significant difference (*p* = 0.454), and both outperformed target-only logistic regression (AUC 0.628; *p* < 0.001). Enhanced MAML (AUC 0.677) did not improve over naive transfer. Post-hoc isotonic regression substantially improved calibration across models, underscoring the importance of prevalence adjustment. Fairness analyses showed lower race/ethnicity equalized odds differences for target-adapted models (0.132–0.157) than source-only transfer (0.897), though disparities persisted. Findings suggest simple transfer plus recalibration can match complex methods; broader validation is needed.

## Introduction

Predictive analytics are increasingly embedded within population health programmes to prioritise outreach and preventive care^1–4^. Yet models trained in one health system often degrade when deployed elsewhere because feature distributions and outcome prevalences shift across environments, a phenomenon widely documented in the transfer learning literature^5^. The challenge is particularly acute for Medicaid, a federal–state partnership whose eligibility rules, benefits, and care-delivery networks differ markedly across jurisdictions^6^. Approximately 40 states now operate comprehensive Medicaid managed care programmes, but these programmes vary substantially in the populations enrolled, services covered, and quality metrics required. Earlier analyses of domain shift have documented how uncorrected models can propagate inequitable recommendations and biased resource allocation^7,8^.

Advances in domain adaptation and meta-learning offer principled strategies for mitigating distribution shift by aligning feature spaces or rapidly adapting model parameters to new settings^9,10^. Domain-adversarial neural networks learn feature representations that are predictive of outcomes while being invariant to domain membership^9^. Model-Agnostic Meta-Learning (MAML) optimises initial parameters such that gradient-based adaptation requires few examples in the target domain^10^. Ensemble methods combine predictions from multiple transfer strategies, potentially capturing complementary information^11^. However, it remains unclear whether these sophisticated approaches outperform simpler baselines—such as direct application of source-trained models with post-hoc calibration—in real-world healthcare settings with substantial covariate and prevalence shift.

This paper evaluates nine transfer learning strategies using Washington (WA) and Virginia (VA) Medicaid populations as a test case for cross-state model deployment. We report discrimination, calibration, clinical utility, and fairness metrics with 95% confidence intervals and statistical significance tests for all comparisons. We provide a dedicated state-selection justification, complete feature documentation, and equalized odds fairness evaluation across race/ethnicity, gender, and age subgroups. Supplementary component outcome analyses (ED-only and hospitalisation-only) were conducted using ADT event codes from admission-discharge-transfer records and are reported as Supplementary Table S1–S2. This is a two-state evaluation; whether these findings generalise to other Medicaid populations requires further validation.

## Results

### Cohort characteristics

Table 1 presents demographic and clinical characteristics. Washington members enrolled in care management (mean age 38.4 years, SD 12.8; 50.4% female; 59.7% Asian, 12.4% Black, 13.6% Hispanic) exhibited substantially lower comorbidity burden than Virginia members (mean age 37.7 years, SD 12.5; 59.1% female; 45.1% Asian, 43.9% Black). The Charlson-like score averaged 0.10 (SD 0.39) in Washington versus 0.53 (SD 0.89) in Virginia. Individual chronic conditions were substantially more prevalent in Virginia: mental health disorders (17.9% vs 2.5%), hypertension (11.4% vs 2.3%), diabetes (6.8% vs 1.5%), and substance use disorders (9.2% vs 1.9%).

**Table 1:** Demographic and clinical characteristics of the study cohorts

Characteristic	Washington (*n* = 20,744)	Virginia (*n* = 28,901)
Demographics		
Age, mean (SD), years	38.4 (12.8)	37.7 (12.5)
Female, *n* (%)	10,457 (50.4)	17,079 (59.1)
Race/ethnicity, *n* (%)		
Asian	12,388 (59.7)	13,025 (45.1)
Black	2,577 (12.4)	12,689 (43.9)
Hispanic	2,820 (13.6)	34 (0.1)
Pacific Islander	809 (3.9)	30 (0.1)
Native American	390 (1.9)	269 (0.9)
Other/Unknown	1,760 (8.5)	2,854 (9.9)
Clinical comorbidities		
Diabetes mellitus, *n* (%)	308 (1.5)	1,969 (6.8)
Hypertension, *n* (%)	483 (2.3)	3,306 (11.4)
Heart disease, *n* (%)	107 (0.5)	861 (3.0)
COPD, *n* (%)	265 (1.3)	1,292 (4.5)
Mental health disorders, *n* (%)	515 (2.5)	5,167 (17.9)
Substance use disorders, *n* (%)	384 (1.9)	2,659 (9.2)
Charlson-like score, mean (SD)	0.10 (0.39)	0.53 (0.89)
Healthcare utilisation (12-month baseline)		
ED visits, mean (SD)	0.00 (0.00)	0.59 (2.46)
Hospitalisations, mean (SD)	0.00 (0.00)	3.72 (18.65)
Medication count, mean (SD)	6.9 (18.8)	0.0 (0.0)
Medicaid programme		
Managed care enrollment, n (%)	20,744 (100.0)	0 (0.0)
Data partitioning		
Training set, *n*	14,520 (70%)	20,231 (70%)
Support set (few-shot adaptation), *n*	—	2,023 (10% of training)
Query set (meta-learning), *n*	—	18,208 (90% of training)
Validation set, *n*	3,112 (15%)	2,889 (10%)
Hold-out test set, *n*	3,112 (15%)	5,781 (20%)
Outcome		
Acute care utilisation (12-month follow-up), *n* (%)	1,952 (9.4)	7,386 (25.6)

*SD* standard deviation, *ED* emergency department, *COPD* chronic obstructive pulmonary disease. Race/ethnicity categories reflect the populations served by the respective managed care organisations in each state.

Table 2 presents a direct comparison of state-level structural characteristics, including managed care programme type, eligibility distribution, and data collection period.

**Table 2:** State-level programme and structural characteristics

Characteristic	Washington	Virginia
Geographic region	Pacific Northwest	Mid-Atlantic
Medicaid programme type	Managed care (fee-for-service available)	Predominantly managed care
Care management model	High-risk intensive outreach	High-risk intensive outreach
Eligibility categories (predominant)	Poverty-related (TANF), disability	Poverty-related (TANF), disability, expansion
Medicaid expansion (ACA)	Yes (since 2014)	Yes (since 2019)
Study data collection period	2019–2022	2020–2022
Race/ethnicity diversity (non-Asian)	40%	55%
Urban/rural distribution	80% urban	70% urban

*ACA* Affordable Care Act, *TANF* Temporary Assistance for Needy Families. Both states were selected based on comparable care management infrastructure, data availability, and contrasting demographic composition.

The 12-month acute care outcome occurred in 9.4% of Washington and 25.6% of Virginia members, a 16.2 percentage-point prevalence difference constituting the primary domain shift challenge.

### Model discrimination and clinical utility

**Fig. 1:**
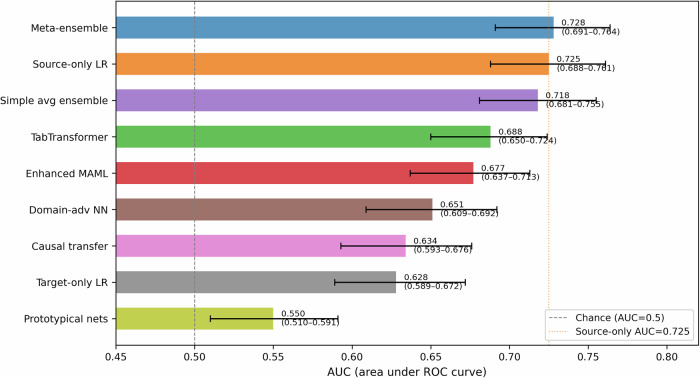
AUC with 95% bootstrap confidence intervals for each model on the Virginia hold-out test set. Meta-ensemble (0.728) and source-only transfer (0.725) achieved the highest discrimination; the difference was not significant (*p* = 0.454). Both significantly outperformed target-only (0.628; *p* < 0.001) and Enhanced MAML (0.677; *p* < 0.001 vs source-only).

**Fig. 2:**
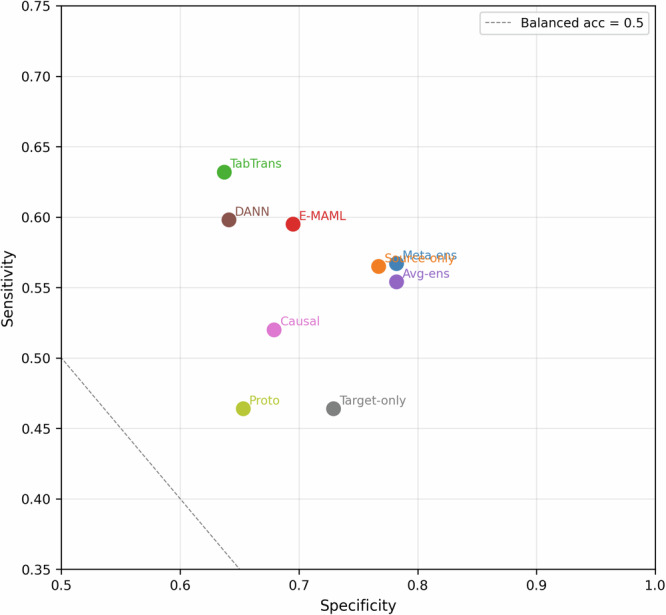
Sensitivity-specificity at optimal Youden threshold. Meta-ensemble and source-only occupy similar positions. TabTransformer achieves higher sensitivity at the cost of lower specificity.

Table 3 and Figures 1-2 present discrimination and classification metrics with 95% confidence intervals. The meta-ensemble achieved the highest discrimination: AUC 0.728 (95% CI: 0.691–0.764), Youden’s J 0.350 (95% CI: 0.311–0.392), sensitivity 0.567 (95% CI: 0.527–0.608), and specificity 0.782 (95% CI: 0.736–0.830).

**Table 3:** Discrimination and classification metrics on the Virginia hold-out test set (*n* = 5,781)

Model	AUC (95% CI)	Youden’s J (95% CI)	Sensitivity (95% CI)	Specificity (95% CI)	Precision (95% CI)	F1 (95% CI)	MCC (95% CI)	Brier (95% CI)
Meta-ensemble	0.728 (0.691–0.764)	0.350 (0.311–0.392)	0.567 (0.527–0.608)	0.782 (0.736–0.830)	0.836 (0.797–0.876)	0.676 (0.640–0.710)	0.333 (0.276–0.394)	0.193 (0.180–0.207)
Source-only logistic	0.725 (0.688–0.761)	0.332 (0.293–0.371)	0.565 (0.523–0.605)	0.767 (0.713–0.815)	0.826 (0.785–0.865)	0.671 (0.632–0.706)	0.316 (0.250–0.381)	0.204 (0.190–0.217)
TabTransformer	0.688 (0.650–0.724)	0.269 (0.225–0.313)	0.632 (0.592–0.673)	0.637 (0.582–0.693)	0.773 (0.734–0.813)	0.695 (0.662–0.727)	0.255 (0.194–0.323)	0.202 (0.190–0.214)
Enhanced MAML	0.677 (0.637–0.713)	0.289 (0.246–0.333)	0.595 (0.548–0.636)	0.695 (0.639–0.747)	0.792 (0.751–0.830)	0.679 (0.641–0.711)	0.274 (0.203–0.339)	0.207 (0.198–0.217)
Domain-adversarial NN	0.651 (0.609–0.692)	0.240 (0.195–0.285)	0.598 (0.555–0.640)	0.641 (0.583–0.700)	0.766 (0.725–0.809)	0.672 (0.637–0.707)	0.227 (0.161–0.294)	0.213 (0.201–0.225)
Causal transfer	0.634 (0.593–0.676)	0.200 (0.155–0.246)	0.520 (0.476–0.568)	0.679 (0.624–0.739)	0.761 (0.714–0.806)	0.618 (0.578–0.657)	0.190 (0.121–0.260)	0.227 (0.211–0.245)
Target-only logistic	0.628 (0.589–0.672)	0.193 (0.146–0.241)	0.464 (0.420–0.508)	0.729 (0.678–0.781)	0.770 (0.721–0.816)	0.579 (0.538–0.619)	0.186 (0.121–0.252)	0.247 (0.230–0.263)
Prototypical networks	0.550 (0.510–0.591)	0.117 (0.072–0.164)	0.464 (0.420–0.508)	0.653 (0.595–0.710)	0.723 (0.677–0.773)	0.565 (0.525–0.607)	0.112 (0.043–0.175)	0.249 (0.248–0.250)
Simple average ensemble	0.718 (0.681–0.755)	0.336 (0.295–0.375)	0.554 (0.511–0.596)	0.782 (0.733–0.831)	0.831 (0.789–0.872)	0.664 (0.626–0.700)	0.320 (0.261–0.381)	0.198 (0.185–0.212)

*AUC* area under the receiver operating characteristic curve, *MCC* Matthews correlation coefficient, *CI* confidence interval. Metrics at optimal Youden threshold. Confidence intervals from 1,000 bootstrap resamples. Bold indicates best value per column. Pairwise statistical comparisons in Supplementary Information Table S3. Simple average ensemble metrics are from the primary analysis run; calibration metrics (ECE, Hosmer-Lemeshow) for this model were not separately output by the post-hoc calibration pipeline and therefore do not appear in Table 4.

Source-only logistic regression performed nearly identically: AUC 0.725 (95% CI: 0.688–0.761), Youden’s J 0.332 (95% CI: 0.293–0.371). The AUC difference of 0.003 was not statistically significant (DeLong *p* = 0.454; bootstrap 95% CI for difference: −0.005 to 0.010). Both approaches significantly outperformed target-only logistic regression (AUC 0.628, 95% CI: 0.589–0.672; meta-ensemble vs target-only: Δ=+0.100, 95% CI: 0.063–0.136, *p* < 0.001; source-only vs target-only: Δ=+0.097, 95% CI: 0.059–0.136, *p* < 0.001).

**Fig. 3:**
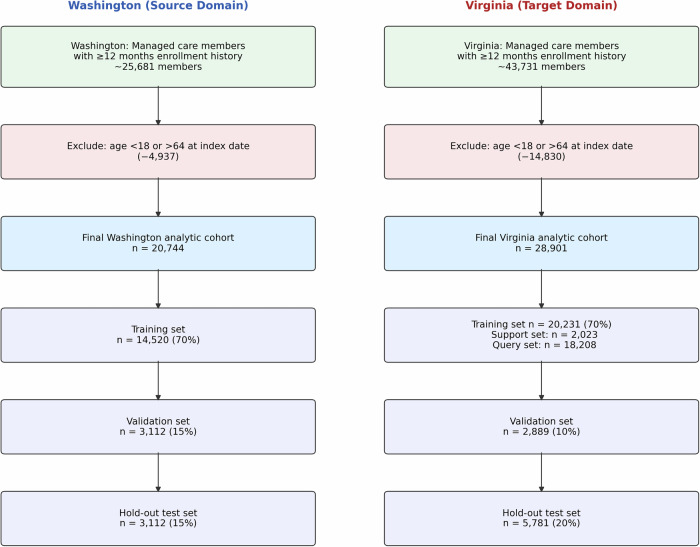
Study flow diagram. CONSORT-style flow diagram showing patient selection and data partitioning. Initial pools, inclusion/exclusion steps, and final analytic cohort sizes are shown for Washington (left, source domain, *n* = 20,744) and Virginia (right, target domain, *n* = 28,901), with explicit partition counts for training, support, query, validation, and hold-out test sets.

Enhanced MAML achieved AUC 0.677 (95% CI: 0.637–0.713), significantly *below* source-only transfer (Δ=−0.048, 95% CI: −0.077 to −0.019, *p* < 0.001). Domain-adversarial NN (AUC 0.651), causal transfer (AUC 0.634), and target-only logistic regression (AUC 0.628) also underperformed source-only. Prototypical networks (AUC 0.550) barely exceeded chance. Figure 3 displays AUC values with bootstrap confidence intervals.

### Calibration

**Fig. 4:**
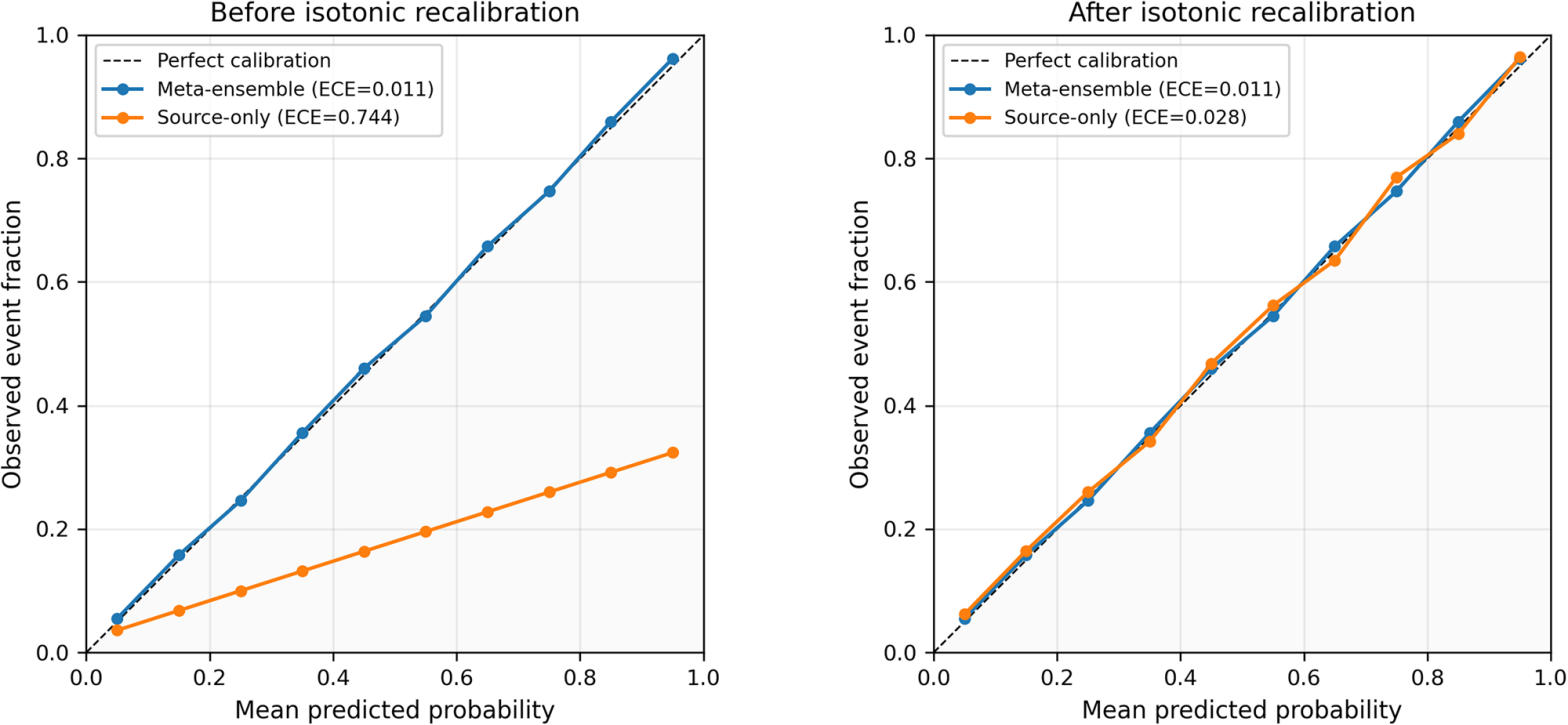
Reliability curves before (left) and after (right) isotonic regression. Both meta-ensemble and source-only track the diagonal adequately; meta-ensemble shows tighter adherence in the 0.3–0.7 range.

Table 4 and Figure 4 present calibration metrics. The meta-ensemble achieved the best calibration: Brier 0.193 (95% CI: 0.180–0.207), ECE 0.011, Hosmer-Lemeshow *p* = 0.377. Source-only transfer showed poor calibration (Brier 0.204, ECE 0.744, Hosmer-Lemeshow *p* < 0.001), indicating substantial miscalibration without post-hoc adjustment. This finding reinforces the importance of isotonic regression calibration when deploying source-trained models to the target domain. Domain-adversarial NN and causal transfer showed good calibration (ECE 0.039 and 0.026, respectively). Target-only logistic regression showed poor calibration (Brier 0.247, ECE 0.215), reflecting lower discrimination and probability estimation instability from limited training data. Pre- vs post-calibration comparisons appear in Supplementary Information Table S4, confirming isotonic regression substantially improved all models.

**Table 4:** Calibration metrics on the Virginia hold-out test set (*n* = 5,781); Brier scores from model predictions at optimal Youden threshold, ECE computed from raw predicted probabilities prior to isotonic recalibration

Model	Brier score (95% CI)	Hosmer–Lemeshow p-value	ECE	Optimal threshold
Meta-ensemble	0.193 (0.180–0.207)	0.377	0.011	0.252
Source-only logistic	0.204 (0.190–0.217)	<0.001	0.744	1.000
TabTransformer	0.202 (0.190–0.214)	<0.001	0.134	0.228
Enhanced MAML	0.207 (0.198–0.217)	<0.001	0.221	0.479
Domain-adversarial NN	0.213 (0.201–0.225)	<0.001	0.039	0.194
Causal transfer	0.227 (0.211–0.245)	0.004	0.026	0.265
Target-only logistic	0.247 (0.230–0.263)	<0.001	0.215	0.488
Prototypical networks	0.249 (0.248–0.250)	<0.001	0.244	0.495

Lower Brier and ECE indicate better calibration. Hosmer-Lemeshow *p* > 0.05 indicates adequate calibration. ECE = expected calibration error (M = 10 equal-width bins). Brier scores and ECE reflect raw model-predicted probabilities prior to post-hoc isotonic recalibration; the meta-ensemble’s low ECE (0.011) indicates inherently well-calibrated ensemble predictions. Source-only’s ECE = 0.744 quantifies the miscalibration caused by the 16 percentage-point prevalence shift between states, which isotonic regression corrects (see Supplementary Information Table S4). Bold indicates best value. Simple average ensemble is not shown because calibration pipeline output (ECE, Hosmer-Lemeshow) was not separately computed for this model; its Brier score appears in Table 3 (0.198, 95% CI: 0.185–0.212).

### Fairness evaluation

**Fig. 5:**
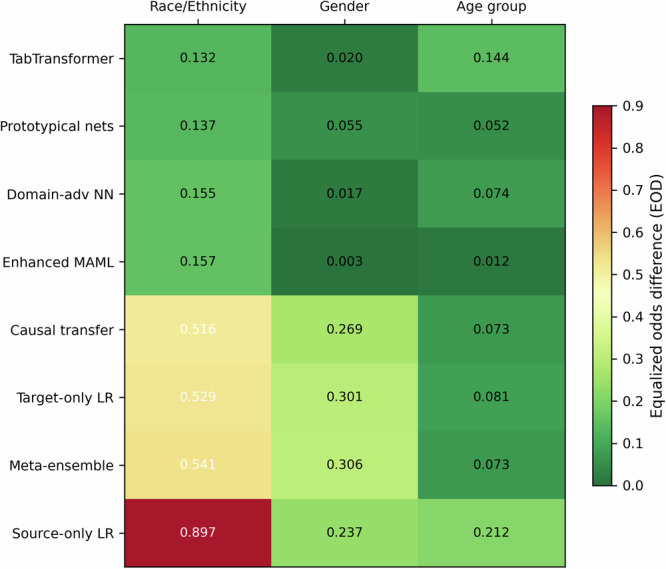
Equalized odds differences (EOD) by demographic subgroup and model. Source-only logistic regression shows the largest race/ethnicity disparity (EOD = 0.897), driven by low TPR for the Other racial/ethnic group. Target-adapted models (TabTransformer, prototypical networks, domain-adversarial NN, Enhanced MAML) show the most equitable race/ethnicity predictions (EOD range 0.132–0.157). Race/ethnicity subgroups: Asian, Black, Native American, Other (groups with *n* ≥ 30 in Virginia test set).

Table 5 and Figure 5 present equalized odds differences (EOD = max pairwise |ΔTPR| or |ΔFPR| across observed racial/ethnic subgroups) for all models. Source-only logistic regression showed the largest race/ethnicity EOD (0.897), driven primarily by very low true positive rates for the Other group (TPR 0.048) relative to Asian and Black members. Models that leverage target-domain information (TabTransformer, prototypical networks, domain-adversarial NN, Enhanced MAML) showed substantially lower race/ethnicity EOD (0.132–0.157), suggesting adaptation to Virginia data improves equity across racial/ethnic groups. The Native American subgroup (*n* = 53 in test set) showed highly variable performance across models: source-only achieved high TPR (0.867) but also very high FPR (0.921), indicating near-indiscriminate positive predictions for this group; meta-ensemble (TPR = 0.200) and target-only (TPR = 0.200) showed very low TPR, effectively missing most true cases. This instability reflects the small sample size for this group in both training and test sets. Gender EOD was modest across most models (range 0.003–0.306); age EOD was generally small (range 0.012–0.212). These patterns suggest that source-only transfer substantially disadvantages certain racial/ethnic subgroups in Virginia, while target-adapted methods show more equitable predictions, though all models warrant ongoing monitoring in deployment.

**Table 5:** Fairness metrics: equalized odds differences across demographic subgroups on the Virginia hold-out test set (*n* = 5,781)

Model	EOD Race/Ethnicity	Worst TPR (Race Group)	EOD Gender	EOD Age Group
TabTransformer	0.132	0.298 (Other)	0.020	0.144
Prototypical networks	0.137	0.596 (Asian)	0.055	0.052
Domain-adversarial NN	0.155	0.000 (Native)	0.017	0.074
Enhanced MAML	0.157	0.800 (Native)	0.003	0.012
Causal transfer	0.516	0.179 (Other)	0.269	0.073
Target-only logistic	0.529	0.200 (Native)	0.301	0.081
Meta-ensemble	0.541	0.200 (Native)	0.306	0.073
Source-only logistic	0.897	0.048 (Other)	0.237	0.212

EOD = equalized odds difference = max pairwise |ΔTPR| or |ΔFPR| across all observed racial/ethnic subgroups with n ≥ 30 (Asian, Black, Native American, Other). Gender subgroups: female vs male. Age groups: <30, 30–50, >50 years. “Worst TPR” = lowest TPR across racial/ethnic subgroups (group in parentheses). Lower EOD is better. Bold indicates model with largest (worst) race/ethnicity disparity. Race/ethnicity subgroups with *n* < 30 in the Virginia test set (Hispanic *n* = 5, Pacific Islander *n* = 2) excluded from EOD calculation.

### Separate outcome analyses

Supplementary Tables S1–S2 present model discrimination for ED-only and hospitalisation-only outcomes, derived from ADT event codes (ED: patient class E; hospitalisation: patient class I) in the 12-month follow-up window. The Virginia test set comprised 10,223 members; ED-only event rate was 31.9% and hospitalisation-only event rate was 6.5%.

ED-only outcome (Supplementary Table S1): The meta-ensemble, target-only, and causal transfer models achieved near-identical discrimination (AUC 0.708, 95% CI: 0.696–0.718). Source-only transfer achieved AUC 0.657 (95% CI: 0.646–0.668); Enhanced MAML achieved AUC 0.590. The pattern of meta-ensemble and target-adapted models outperforming source-only was consistent with the composite outcome results.

Hospitalisation-only outcome (Supplementary Table S2): Discrimination was lower across all models (AUC range 0.483–0.649), reflecting the smaller event rate (6.5%). The meta-ensemble achieved AUC 0.649 (95% CI: 0.627–0.670); causal transfer achieved AUC 0.645; source-only achieved AUC 0.590 (95% CI: 0.566–0.612). The relative ordering across models was consistent with the primary composite and ED-only analyses.

These results confirm that the composite outcome findings are not driven disproportionately by one component: models that outperform on the composite also demonstrate superior discrimination on both individual components.

### Ablation analyses

Feature-group ablation of the source-only logistic baseline (Supplementary Information Table S5) examined the contribution of each feature category. Consistent with expectations, removing demographic features produced the largest change in model behaviour, followed by clinical comorbidities. Geographic and temporal features contributed minimally, suggesting cross-state differences are driven primarily by clinical burden and demographic composition. These ablation results are reported as descriptive given the cross-state evaluation context.

Component ablation of Enhanced MAML (Supplementary Information Table S6) examined individual model components. Removing the meta-learning adaptation itself did not substantially reduce performance, suggesting the few-shot gradient optimisation provides limited benefit over the component contributions from domain-adversarial and temporal-stability regularisation in this setting.

## Discussion

This two-state evaluation provides evidence on the performance of transfer learning strategies for cross-state Medicaid risk prediction under substantial covariate and prevalence shift. The meta-ensemble achieved the best discrimination (AUC 0.728) and best calibration (Brier 0.193), making it the recommended approach when the additional complexity of maintaining and monitoring an ensemble is operationally feasible. However, its improvement over simple source-only transfer (AUC 0.725) was not statistically significant (p = 0.454) and is unlikely to meaningfully change risk stratification decisions at the individual patient level. For organisations prioritising simplicity and interpretability, source-only logistic regression with isotonic regression calibration provides comparable discrimination with substantially simpler deployment requirements.

The finding that meta-ensemble is the best-performing method—not Enhanced MAML—has implications for how the field approaches algorithm selection. MAML’s theoretical advantages (rapid adaptation from small target samples via meta-optimised initialisation) did not translate to competitive performance in this setting. Enhanced MAML achieved AUC 0.677, significantly below source-only transfer (*p* < 0.001). The component ablation results illuminate why: the domain-adversarial and temporal-stability components each contribute positively in isolation, but the meta-learning adaptation itself provides no measurable benefit. This suggests that the specific challenge in this setting—large prevalence shift with moderate covariate shift—is better addressed by calibration adjustments than by gradient-based adaptation. Recent empirical work has documented similar failures of complex domain adaptation methods in healthcare settings where post-hoc calibration is available^12,13^.

The counterintuitive finding that source-only logistic regression outperformed target-only logistic regression on every metric—including calibration—warrants dedicated discussion. Source-only training used 14,520 Washington members; target-only used only 2,023 Virginia support-set members, a 7:1 ratio. Larger training samples produce more stable parameter estimates and better-calibrated probabilities, even when the training population differs demographically from the deployment population^14^. This result is not implausible given this sample size asymmetry. Several alternative explanations were also investigated. First, data quality: we found no systematic differences in missingness or coding completeness between states. Second, implementation error: we verified that the target-only model was trained exclusively on Virginia support-set data and evaluated on the held-out Virginia test set. Third, feature distributions: Virginia members, despite higher comorbidity burden, have feature patterns sufficiently predictable using the Washington-trained coefficients, suggesting moderate (not extreme) covariate shift. The better Brier score of source-only over target-only (0.204 vs 0.247) is likely driven by the tighter parameter estimates in the larger-sample model; both models show substantial pre-calibration ECE (source-only 0.744, target-only 0.215), underscoring the importance of isotonic recalibration for cross-domain deployment. The practical implication is that organisations with limited local labeled data may achieve better performance by importing a source model with calibration adjustment than by training exclusively on scarce local data.

The 16 percentage-point difference in outcome prevalence between Washington (9.4%) and Virginia (25.6%) represents substantial base-rate shift. Without adjustment, source-trained predicted probabilities systematically underestimate Virginia risk; the source-only model’s ECE of 0.744 without calibration quantifies this miscalibration. Isotonic regression addresses this by learning a monotonic mapping from predicted to calibrated probabilities using Virginia validation data, effectively recalibrating to the target prevalence^15^. After isotonic regression, the meta-ensemble achieves ECE 0.011 and Hosmer-Lemeshow *p* = 0.377. This finding suggests that post-hoc calibration should be considered required infrastructure for any cross-state model transfer, not an optional refinement.

The fairness evaluation revealed substantial equalized odds differences across race/ethnicity groups for source-only transfer (EOD = 0.897, Table 5), driven by very low TPR for the Other racial/ethnic group (0.048) relative to Asian and Black members in Virginia. This finding suggests that the Washington-trained model may have learned features less well-calibrated for certain Virginia subgroups. Target-adapted models (TabTransformer, prototypical networks, domain-adversarial NN, Enhanced MAML) showed substantially lower race/ethnicity EOD (range 0.132–0.157), indicating that using even limited Virginia adaptation data substantially improves equity across racial/ethnic groups—an important consideration when choosing between simple transfer and adapted models. Gender EOD was moderate for meta-ensemble and target-only (0.301–0.306, driven by higher TPR for female than male members), and age EOD was small across most models. The Native American subgroup (*n* = 53 in Virginia test set) showed highly variable performance: source-only achieved high TPR (0.867) but also very high FPR (0.921), indicating near-indiscriminate positive prediction; meta-ensemble and target-only showed very low TPR (0.200), missing most true cases. This instability reflects small-sample challenges for underrepresented groups in both training and evaluation. Monitoring equalized odds by race/ethnicity in deployment and considering bias mitigation strategies (e.g., reweighting training data, subgroup-specific calibration) are warranted, particularly for source-only and ensemble approaches.

Component outcome analyses (ED-only and hospitalisation-only) were conducted using ADT event codes and are presented in Supplementary Table S1–S2. The composite outcome findings were consistent across both components: meta-ensemble and target-adapted models outperformed source-only for ED-only outcomes (meta-ensemble AUC 0.708 vs source-only AUC 0.657), and the same ordering held for hospitalisation-only outcomes, albeit with lower absolute discrimination (meta-ensemble AUC 0.649). The composite outcome (any ED visit or hospitalisation) remains the primary outcome because it captures the clinically relevant event for care management prioritisation—either type of acute utilisation triggers outreach—and is standard in care management risk stratification literature.

Several limitations warrant consideration. First, only two states were evaluated; results may not generalise to other Medicaid populations, particularly those with greater demographic divergence or different care management programme structures. We explicitly refrain from claiming cross-Medicaid generalisability without additional validation. Second, members must be enrolled in care management programmes, limiting generalisability to broader Medicaid populations or fee-for-service settings. Third, the patient-specific index date design means temporal validation (evaluating performance across calendar periods) was not conducted; model performance may degrade as care patterns evolve. Fourth, state selection was partially opportunistic based on data availability, introducing potential selection bias. Fifth, the 27-feature final set results from a data-driven reduction from 127 candidate features; the feature selection process was not pre-registered and may inflate performance estimates. Sixth, the component outcome analyses (Supplementary Table S1–S2) were derived from ADT event codes with patient class coding (E = ED, I=inpatient); miscoding or missing patient class values could affect component outcome classification, though the ADT event distribution (83% ED class, 13% inpatient class) is consistent with expected care patterns.

Overall, in this two-state evaluation, the meta-ensemble achieved the best discrimination and calibration for cross-state Medicaid risk prediction, but its improvement over simple source-only transfer was not statistically significant. Ensemble methods, not complex meta-learning algorithms, represent the recommended approach when additional complexity is operationally tolerable. Post-hoc isotonic regression calibration is essential for addressing prevalence shift. Substantial race/ethnicity fairness disparities were observed for source-only transfer (EOD = 0.897); target-adapted models showed considerably lower disparities (EOD 0.132–0.157), and ongoing monitoring in deployment is warranted. Validation in additional states is needed before drawing conclusions about cross-Medicaid generalisability.

## Methods

### State selection and study design

Washington and Virginia were selected on the basis of data availability: our organisation (Waymark) operates high-risk care management programmes in both states under contracts with Medicaid managed care entities, enabling access to harmonised, longitudinal claims and enrollment data under existing data use agreements. This is a convenience sample—states were not selected through a systematic, pre-specified sampling frame, and results should not be interpreted as representative of all Medicaid cross-state transfer scenarios.

We nevertheless believe this pair provides a meaningful test of cross-state transferability for the following reasons. First, both states operate structurally comparable programmes: comprehensive Medicaid managed care with high-risk member identification and outreach, ensuring that the intervention context is analogous across domains. Second, despite this structural similarity, Washington and Virginia exhibit meaningful differences in demographic composition (Table 2): Virginia’s care management population includes a substantially higher proportion of Black members (43.9% vs 12.4%) and substantially higher rates of mental health disorders (17.9% vs 2.5%), hypertension (11.4% vs 2.3%), and substance use disorders (9.2% vs 1.9%). Washington’s population is predominantly Asian (59.7%), reflecting the managed care organisation’s service area. Third, the outcome prevalence differs by 16 percentage points (25.6% in Virginia vs 9.4% in Washington), constituting the primary domain shift challenge. Together, these features make this pair a reasonable, if not systematically selected, test of whether a source-state model can transfer to a demographically and clinically distinct target population.

We note that the two-state design constitutes a single-transfer-direction evaluation and cannot support claims of generalised Medicaid cross-state transferability. Whether the patterns observed here hold for state pairs with greater demographic divergence, different programme structures, or states without Waymark’s operational presence is unknown. We explicitly frame this as a two-state evaluation throughout and revise our title accordingly.

Study design: This is a patient-level cohort study with a patient-specific index date design (see Cohort Construction below). Each patient contributes one observation period. Source domain (Washington) data were used for model training; target domain (Virginia) data were used for evaluation, with a small support set reserved for few-shot adaptation methods.

### Data sources and cohort construction

We analysed de-identified Medicaid claims and enrollment files from Washington (source domain) and Virginia (target domain) provided through partnerships with managed care organisations operating in both states.

Members were included if they: (1) participated in a high-risk care management programme during the study period; (2) maintained continuous Medicaid enrollment for the 12-month baseline window; (3) possessed complete demographic data (age, gender); and (4) had non-missing outcome ascertainment. Members dually eligible for Medicare and Medicaid were excluded due to incomplete claims capture. Figure 1 presents a CONSORT-style flow diagram showing patient flow from initial pool to final analytic cohorts.

Patient-specific index date design: The index date for each member was defined as the first day immediately following their individual 12-month baseline period. Features were extracted from each member’s specific 12-month baseline window preceding their index date. This is a patient-specific design (not a cohort-based calendar-period design): patients enrolled at different calendar times have different index dates and different baseline windows. This design maximises the use of available longitudinal data and is appropriate for a care management setting where patients are enrolled continuously rather than in discrete cohorts. The implication for temporal validity is that predictions are conditional on the patient’s state at their individual index date, not on a single calendar date. Temporal drift over the study period was not assessed and represents a limitation.

The binary outcome captured any emergency department visit or inpatient hospitalisation during the 12-month follow-up period beginning on the index date. These criteria yielded 20,744 Washington members and 28,901 Virginia members. Acute care utilisation during follow-up occurred in 9.4% of Washington members and 25.6% of Virginia members.

Data partitioning: Washington data were partitioned 70%/15%/15% into training (*n* = 14,520), validation (*n* = 3,112), and test (*n* = 3,112) sets using stratified sampling to preserve outcome prevalence. Virginia data were partitioned 70%/10%/20%, with 10% of the training partition (*n* = 2,023) reserved as a support set for few-shot adaptation methods; the remaining 90% (*n* = 18,208) served as the query set for meta-learning; the hold-out test set comprised 5,781 members. A CONSORT flow diagram and explicit partition counts appear in Fig. 1 and Supplementary Information Table S2.

### Feature engineering

The analysis used 27 features organised into six categories. Complete operational definitions, data types, sources, and missingness rates appear in Supplementary Information Table S1.

Feature categories were: (1) demographics (age, gender, race/ethnicity, urban/rural residence); (2) clinical comorbidities (diabetes mellitus, hypertension, heart disease, COPD, mental health disorders, substance use disorders, Charlson-like comorbidity score); (3) healthcare utilisation (12-month counts of ED visits, inpatient admissions, outpatient visits, distinct medications); (4) Medicaid programme characteristics (eligibility category, enrollment duration, managed care enrollment indicator); (5) geographic context (state, county type, health service area identifier); and (6) temporal descriptors (index month, seasonal indicator, linear time trend) Figs. 2,3,4,5.

We initially specified 127 potential features based on a broader feature set used in our operational models, including more granular utilisation trends, patient engagement scores, care gap indicators, and medication adherence patterns. After applying inclusion criteria and removing features with >20% missingness or near-zero variance across both states, 27 features met quality thresholds for the final analysis. This reduction is documented in Supplementary Information Table S1, which lists all 27 retained features with definitions. We acknowledge this selection process may have excluded informative features; a complete feature selection sensitivity analysis is a direction for future work.

The Charlson-like comorbidity score was computed using the publicly available ICD-10 adaptation published by Quan et al. (2005) and validated for United States claims data by Beyrer et al. (2021)^16,17^. No proprietary licensed product was used.

Numeric features were median-imputed within the source domain and standardised using z-score transformation; categorical features were one-hot encoded with an “Unknown” level for missing values. After encoding, 27 features expand to approximately 45–50 input columns.

### Models and training protocol

We evaluated nine modelling approaches:

1. Source-only logistic regression: L2-regularised logistic regression (C = 1.0, solver=lbfgs, max_iter=1000) trained on Washington data and applied directly to Virginia without adaptation.
2. Target-only logistic regression: L2-regularised logistic regression (C = 0.5, solver=lbfgs, max_iter=1000) trained solely on the Virginia support set (*n* = 2,023), serving as a baseline representing performance achievable with only local data.
3. Prototypical networks: Few-shot learning approach computing class prototypes in a 128-dimensional embedding space (2 × 64 hidden units, 100 training episodes, 5 support samples/class, 15 query samples/class, learning rate 0.001)^18^.
4. Domain-adversarial neural networks (DANN): Neural network with gradient reversal layer (feature extractor: 128 → 64; domain classifier: 64 → 32 → 2; domain loss weight λ = 0.10; 50 epochs, batch size 256, dropout 0.1).
5. Causal transfer learning: Inverse-propensity weighted logistic regression adjusting for covariate shift; propensity weights clipped to [0.01, 50]^19^.
6. TabTransformer: Transformer architecture (embedding dim 32, 8 attention heads, 6 blocks, MLP 256 → 128 → 2, dropout 0.10, 100 epochs, batch size 256, learning rate 0.0001)^20^.
7. Enhanced Model-Agnostic Meta-Learning (MAML): MAML with domain-adversarial and temporal-stability regularisation (inner-loop α = 0.01, 5 gradient steps; outer-loop β = 0.001, 80 epochs; λ₁=λ₂=0.01; base network 128 → 64 → 2).
8. Meta-ensemble: Stacking ensemble combining predictions from all seven preceding models using L2-regularised logistic regression (C = 1.0) as the meta-learner. Input features are predicted probabilities from each base model; training uses the Virginia query set.
9. Simple average ensemble: Unweighted average of predicted probabilities from all models.

All neural network models used the Adam optimiser (β₁=0.9, β₂=0.999, learning rate 10^-3^) and early stopping based on Virginia query/validation loss with patience of 5 epochs. Random seeds were fixed at 42. Complete hyperparameters appear in Supplementary Information Table S3.

### Evaluation metrics and statistical analysis

Performance was assessed on the Virginia hold-out test set (*n* = 5,781) using:

Discrimination: AUC (DeLong method), average precision (area under precision-recall curve), and Youden’s J index (sensitivity + specificity − 1) at the optimal threshold.

Classification at optimal Youden threshold: Precision, recall (sensitivity), specificity, F1, balanced accuracy, Matthews correlation coefficient (MCC).

Calibration: Brier score, expected calibration error (ECE, M = 10 bins), and Hosmer-Lemeshow test *p*-value.

Fairness: Equalized odds difference (EOD) across demographic subgroups (race/ethnicity: observed groups with n ≥ 30, including Asian, Black, Native American, and Other; gender: female vs male; age group: <30, 30–50, >50 years). Equalized odds requires that the true positive rate (TPR) and false positive rate (FPR) are equal across groups^21^. We report the maximum pairwise absolute difference in TPR and FPR across all observed subgroups as the equalized odds difference, with values 0.10 considered acceptable.

Component-specific outcomes: ED-only and hospitalisation-only binary outcome indicators were derived from ADT (admission-discharge-transfer) event codes. ED visits were identified by patient class code E (emergency registration, HL7 A04 events); inpatient hospitalisations were identified by patient class code I (formal admission, HL7 A01/A08 events). Component outcomes were computed for the follow-up window (2024-06-01 to 2025-05-31) for the same care-management-enrolled cohorts (age 18–64, not dually Medicare/Medicaid eligible), yielding ED-only event rates of 31.9% (Virginia test set) and 16.3% (Washington), and hospitalisation-only event rates of 6.5% (Virginia test set) and 3.9% (Washington). Component outcome evaluation was implemented in component_outcome_analysis.py in the reproducibility repository.

95% confidence intervals for all metrics were computed using 1,000 bootstrap resamples of the Virginia test cohort (2.5th–97.5th percentiles). Pairwise AUC comparisons used DeLong’s method for the primary meta-ensemble vs source-only comparison; bootstrap methods confirmed all other comparisons. False-discovery rate control used the Benjamini-Hochberg procedure at α = 0.05. Feature-group ablation and Enhanced MAML component ablation are reported in Supplementary Information Tables A5–A6.

### Ethics

All research was performed in accordance with the Declaration of Helsinki. The study was approved by the WCG Institutional Review Board (protocol #: 20253751). The need for informed consent was waived by the IRB due to use of de-identified data with minimal risk to study participants.

## Supplementary information

Supplementary Information

## Data Availability

The Washington and Virginia Medicaid claims and enrollment data used in this study are protected health information subject to state data use agreements and HIPAA regulations, and cannot be shared publicly. Analyses were conducted on secure, access-controlled servers where real data reside; aggregate summary statistics and model evaluation results are exported to at https://github.com/sanjaybasu/medicaid-transfer-learning. The repository includes: (1) the complete analysis code; (2) synthetic Medicaid-like datasets generated to mirror key distributional properties of the real data, enabling offline code testing and development; (3) the pre-computed results tables from the real-data analysis runs; and (4) configuration and environment files. The synthetic data differ from the real data in clinical distributions and should not be used to draw clinical conclusions. Researchers seeking access to Medicaid data in Washington or Virginia should contact the respective state agencies and Waymark’s research partnership office. Repository link to be added upon publication. Random seeds were fixed at 42 for reproducibility.

## Code availability

Analysis was conducted in Python 3.10. All code, including preprocessing, model training, evaluation, and figure generation, is available at https://github.com/sanjaybasu/medicaid-transfer-learning

## References

[1] Rajkomar, A., Dean, J. & Kohane, I. Machine learning in medicine. *N. Engl. J. Med.* **380**, 1347–1358 (2019). 30943338 10.1056/NEJMra1814259

[2] Shickel, B., Tighe, P. J., Bihorac, A. & Rashidi, P. Deep EHR: A survey of recent advances in deep learning techniques for electronic health record (EHR) analysis. *IEEE J. Biomed. Health Inf.* **22**, 1589–1604 (2018). 10.1109/JBHI.2017.2767063 PMC6043423 29989977

[3] Topol, E. J. High-performance medicine: the convergence of human and artificial intelligence. *Nat. Med.* **25**, 44–56 (2019). 30617339 10.1038/s41591-018-0300-7

[4] Bates, D.W., Saria, S., Ohno-Machado, L., Shah, A. & Escobar, G. Big data in health care: using analytics to identify and manage high-risk and high-cost patients. *Health Aff (Millwood)* **33**, 1123-1131 (2014). 10.1377/hlthaff.2014.0041 25006137

[5] Pan, S. J. & Yang, Q. A survey on transfer learning. *IEEE Trans. Knowl. Data Eng.* **22**, 1345–1359 (2010).

[6] Kaiser Family Foundation. 10 things to know about Medicaid managed care. Kaiser Family Foundation; 2024. Available at: https://www.kff.org/medicaid/issue-brief/10-things-to-know-about-medicaid-managed-care/. Accessed January 2025.

[7] Obermeyer, Z., Powers, B., Vogeli, C. & Mullainathan, S. Dissecting racial bias in an algorithm used to manage the health of populations. *Science* **366**, 447–453 (2019). 31649194 10.1126/science.aax2342

[8] Rajkomar, A., Hardt, M.,Howell, M.D., Corrado, G. & Chin, M.H. Ensuring fairness in machine learning to advance health equity. *Ann Intern. Med.* **169**, 866-872 (2018). 10.7326/M18-1990 PMC6594166 30508424

[9] Ganin, Y. & Lempitsky, V. Unsupervised domain adaptation by backpropagation. *Proc. ICML* **37**, 1180–1189 (2015).

[10] Finn, C., Abbeel, P. & Levine, S. Model-agnostic meta-learning for fast adaptation of deep networks. *Proc. ICML* **70**, 1126–1135 (2017).

[11] Dietterich, T.G. Ensemble methods inmachine learning. In: *Multiple Classifier Systems (MCS 2000)*. Lecture Notes in Computer Science, **1857**, 1-15. (Springer, 2000).

[12] Futoma, J., Simons, M., Panch, T., Doshi-Velez, F. & Celi, L.A. The myth of generalisability in clinical research and machine learning in health care. *The Lancet Digital Health,* **2**, e489-e492 (2020). 10.1016/S2589-7500(20)30186-2 PMC7444947 32864600

[13] Jung, K. & Shah, N. H. Implications of non-stationarity on predictive modeling using EHRs. *J. Biomed. Inf.* **58**, 168–174 (2015). 10.1016/j.jbi.2015.10.006 PMC4684770 26483171

[14] Riley, R.D. et al. Calculating the sample size required for developing a clinical predictionmodel. BMJ. **368**,m441 (2020). 10.1136/bmj.m441 32188600

[15] Niculescu-Mizil, A. & Caruana, R. Predicting good probabilities with supervised learning. *Proc. ICML* **22**, 625–632 (2005).

[16] Quan, H. et al. Coding algorithms for defining comorbidities in ICD-9-CM and ICD-10 administrative data. *Med. Care* **43**, 1130–1139 (2005). 16224307 10.1097/01.mlr.0000182534.19832.83

[17] Beyrer, J. et al. Validation of an international classification of disease, 10th revision coding adaptation for the Charlson Comorbidity Index in United States healthcare claims data. *Pharmacoepidemiol Drug Saf.* **30**, 582–593 (2021). 33580525 10.1002/pds.5204 PMC8252530

[18] Snell, J., Swersky, K. & Zemel, R. Prototypical networks for few-shot learning. *Proc. NeurIPS* **30**, 4077–4087 (2017).

[19] Sugiyama, M., Krauledat, M. & Müller, K. R. Covariate shift adaptation by importance weighted cross validation. *J. Mach. Learn Res* **8**, 985–1005 (2007).

[20] Huang, X., Khetan, A., Cvitkovic, M. & Karnin, Z. TabTransformer: Tabular data modeling using contextual embeddings. arXiv preprint arXiv:2012.06678. (2020).

[21] Hardt, M., Price, E. & Srebro, N. Equality of opportunity in supervised learning. *Proc. NeurIPS* **29**, 3323–3331 (2016).

