# Supplementary material for: Transferring healthcare risk prediction models between Medicaid populations: a transfer learning evaluation
Source: Npj Health Syst. 2026 Jun 19;3:48. doi: 10.1038/s44401-026-00097-w (PMC13354197; doi:10.1038/s44401-026-00097-w)

Supplementary Information

Transferring Healthcare Risk Prediction Models Between Two Medicaid Populations: A Transfer Learning Evaluation

---

Supplement Table S1. Complete feature definitions, data types, sources, and missingness rates (27 retained features).

Features were drawn from a candidate set of 127 operational variables. After applying quality thresholds (missingness <20% in both states, non-zero variance in both states), 27 features were retained. Excluded features (n=100) included patient engagement scores (38–62% missing in VA), care gap indicators (HEDIS-based; near-zero variance in care-management-enrolled subpopulation), granular utilisation trend trajectories, and medication adherence metrics (MPR; >20% missing in WA). WA = Washington; VA = Virginia.

#	Category	Feature Name	Definition	Data Type	Source	Missingness WA	Missingness VA
1	Demographics	age	Age in years at index date	Continuous	Enrollment	0.0%	0.0%
2	Demographics	gender	Sex (Female/	Categorical (3 levels)	Enrollment	0.0%	0.0%

#	Category	Feature Name	Definition	Data Type	Source	Missingness WA	Missingness VA
			Male/Unknown)				
3	Demographics	race_ethnicity	Self-reported: Asian, Black, Hispanic, Native American, Pacific Islander, Other/Unknown	Categorical (6+ levels)	Enrollment	0.0%	0.0%
4	Demographics	urban_rural	Urban/suburban/rural (ZIP-code RUCA classification)	Categorical (4 levels)	Enrollment	1.2%	1.8%
5	Clinical	diabetes_mellitus	ICD-10 E08–E11, E13 in 12-month baseline	Binary	Claims	0.0%	0.0%
6	Clinical	hypertension	ICD-10 I10–I13,	Binary	Claims	0.0%	0.0%

#	Category	Feature Name	Definition	Data Type	Source	Missingness WA	Missingness VA
			I15 in 12-month baseline				
7	Clinical	heart_disease	ICD-10 I20–I25, I50–I51, I70 in 12-month baseline	Binary	Claims	0.0%	0.0%
8	Clinical	copd	ICD-10 J41–J44 in 12-month baseline	Binary	Claims	0.0%	0.0%
9	Clinical	mental_health_disorders	ICD-10 F20–F25, F30–F34, F39 in 12-month baseline	Binary	Claims	0.0%	0.0%
10	Clinical	substance_abuse_disorders	ICD-10 F10–F19 in 12-month baseline	Binary	Claims	0.0%	0.0%

#	Category	Feature Name	Definition	Data Type	Source	Missingness WA	Missingness VA
11	Clinical	charlson_comorbidity_index	Charlson-like score (ICD-10 adaptation per Quan et al. 2005, validated by Beyrer et al. 2021)	Integer (0–10+)	Claims (derived)	0.0%	0.0%
12	Utilisation	prior_ed_visits_12m	Count of ED visits (revenue codes 0450–0459; CPT 99281–99285) in 12-month baseline	Integer	Claims	0.0%	0.0%
13	Utilisation	prior_hospitalizations_12m	Count of inpatient admissions (bill type 11X, 12X) in	Integer	Claims	0.0%	0.0%

#	Category	Feature Name	Definition	Data Type	Source	Missingness WA	Missingness VA
			12-month baseline				
14	Utilisation	prior_outpatient_visits_12m	Count of outpatient E/M visits (CPT 99201–99499) in 12-month baseline	Integer	Claims	0.0%	0.0%
15	Utilisation	medication_count	Count of distinct NDC-level medications dispensed in 12-month baseline	Integer	Pharmacy claims	0.0%	0.0%
16	Medicaid	eligibility_category	Primary eligibility pathway: poverty/TANF, disability, expansion, other	Categorical (4 levels)	Enrollment	0.8%	1.1%

#	Category	Feature Name	Definition	Data Type	Source	Missingness WA	Missingness VA
17	Medicaid	enrollment_duration	Months continuously enrolled at index date	Continuous	Enrollment	0.0%	0.0%
18	Medicaid	managed_care_enrollment	Enrolled in managed care organisation at index date	Binary	Enrollment	0.0%	0.0%
19	Geographic	state	State identifier (WA=0, VA=1); used as domain label for DANN	Binary	Enrollment	0.0%	0.0%
20	Geographic	county_type	USDA Rural-Urban Continuum: metropoli	Categorical (3 levels)	Enrollment/NCHS	1.5%	2.1%

#	Category	Feature Name	Definition	Data Type	Source	Missingness WA	Missingness VA
			tan, micropolitan, rural				
21	Geographic	health_service_area	Dartmouth Atlas HSA identifier for regional clustering	Categorical	Enrollment	3.2%	3.8%
22	Temporal	enrollment_month	Calendar month of index date (1–12)	Integer	Derived	0.0%	0.0%
23	Temporal	season_winter	Index month in Dec–Feb	Binary	Derived	0.0%	0.0%
24	Temporal	season_spring	Index month in Mar–May	Binary	Derived	0.0%	0.0%
25	Temporal	season_summer	Index month in Jun–Aug	Binary	Derived	0.0%	0.0%

#	Category	Feature Name	Definition	Data Type	Source	Missingness WA	Missingness VA
26	Temporal	season_fall	Index month in Sep–Nov	Binary	Derived	0.0%	0.0%
27	Temporal	time_trend	Linear trend: months since study start (January 2019)	Integer	Derived	0.0%	0.0%

Notes: No feature exceeded 4% missingness in either state, ruling out differential data quality as an explanation for cross-state performance differences. Numeric features imputed using source-domain training medians; categorical features assigned an "Unknown" level for missingness. After one-hot encoding, 27 features expand to approximately 45–50 input columns. ICD-10 = International Classification of Diseases, 10th Revision; RUCA = Rural-Urban Commuting Area; NCHS = National Center for Health Statistics; HSA = Health Service Area; NDC = National Drug Code; CPT = Current Procedural Terminology.

Supplement Table S2. CONSORT-style patient flow and data partitioning reconciliation.

Patient Flow

Step	Washington	Virginia
Initial pool (managed care members with ≥12 months enrollment history in study period)	~25,681	~43,731
Exclude: age <18 or >64 years at index date	−4,937	−14,830
Exclude: Medicare dual-eligible (incomplete claims)	Included in above	Included in above
Exclude: missing demographic data	0	0
Exclude: missing outcome data	0	0
Final analytic cohort	20,744	28,901

Data Partitioning

Partition	Washington	Virginia
Training set	14,520 (70%)	20,231 (70%)
— Support set (few-shot adaptation)	—	2,023 (10% of training)

Partition	Washington	Virginia
— Query set (meta-learning)	—	18,208 (90% of training)
Validation set	3,112 (15%)	2,889 (10%)
Hold-out test set	3,112 (15%)	5,781 (20%)
Total	20,744	28,901

Arithmetic verification: WA: 14,520 + 3,112 + 3,112 = 20,744 ✓. VA: 20,231 + 2,889 + 5,781 = 28,901 ✓. VA training: 2,023 + 18,208 = 20,231 ✓. All partitions used stratified sampling preserving outcome prevalence. Outcome prevalence in VA test set: 25.6% (n=1,477 events in 5,781 members). Outcome prevalence in WA test set: 9.4% (n=293 events in 3,112 members).

Reconciliation note: An earlier response letter incorrectly cited initial pool sizes (~51,847 WA; ~69,203 VA) as the analytic cohort sizes. The correct initial pool sizes (after restricting to care management programme enrollees with ≥12 months enrollment history but before applying inclusion/exclusion criteria) were approximately 25,681 WA and 43,731 VA, not 51,847/69,203. The final analytic cohorts after age filtering and exclusions are 20,744 WA and 28,901 VA.

Supplement Table S3. Pairwise AUC comparisons with 95% bootstrap confidence intervals and Benjamini-Hochberg FDR-adjusted p-values (Virginia hold-out test set, n=5,781).

Primary comparison (meta-ensemble vs source-only) additionally verified using DeLong's asymptotic method. All other comparisons use 1,000-bootstrap distribution of pairwise AUC differences. FDR correction applied at α=0.05 across all 14 comparisons.

Comparison	ΔAUC	95% CI	Bootstrap p	FDR-adjusted significant?
Meta-ensemble vs Source-only	+0.003	−0.005 to +0.010	0.454	No
Source-only vs Target-only	+0.097	+0.059 to +0.136	<0.001	Yes
Meta-ensemble vs Target-only	+0.100	+0.063 to +0.136	<0.001	Yes
Source-only vs Enhanced MAML	+0.048	+0.019 to +0.077	<0.001	Yes
Meta-ensemble vs Enhanced MAML	+0.051	+0.022 to +0.082	<0.001	Yes
Source-only vs TabTransformer	+0.037	−0.011 to +0.062	0.060	No
Source-only vs Domain-adversarial NN	+0.074	+0.048 to +0.098	0.008	Yes
Source-only vs Causal transfer	+0.091	+0.060 to +0.122	<0.001	Yes
Source-only vs Prototypical networks	+0.175	+0.141 to +0.210	<0.001	Yes
Target-only vs Causal transfer	−0.006	−0.016 to +0.001	0.692	No

Comparison	ΔAUC	95% CI	Bootstrap p	FDR-adjusted significant?
Target-only vs Prototypical networks	+0.078	+0.043 to +0.112	<0.001	Yes
Meta-ensemble vs Causal transfer	+0.094	+0.061 to +0.126	<0.001	Yes
Meta-ensemble vs Domain-adversarial NN	+0.077	+0.050 to +0.104	<0.001	Yes
Meta-ensemble vs Prototypical networks	+0.178	+0.143 to +0.213	<0.001	Yes

Notes: Positive ΔAUC indicates first-named model superior. Meta-ensemble vs source-only DeLong p=0.452 (confirms bootstrap result). Confidence intervals computed as 2.5th–97.5th percentiles of 1,000 bootstrap resamples of the Virginia test set.

Supplement Table S4. Pre- vs post-isotonic regression Brier scores by model (Virginia hold-out test set, n=5,781).

Model	Pre-calibration Brier	Post-calibration Brier (Platt scaling)	Post-calibration Brier (Isotonic regression)
Meta-ensemble	0.193	0.170	0.172

Model	Pre-calibration Brier	Post-calibration Brier (Platt scaling)	Post-calibration Brier (Isotonic regression)
Source-only logistic	0.204	0.190	0.190
TabTransformer	0.202	0.190	0.191
Enhanced MAML	0.207	0.190	0.190
Domain-adversarial NN	0.213	0.190	0.191
Causal transfer	0.227	0.171	0.171
Target-only logistic	0.247	0.170	0.171
Prototypical networks	0.249	0.190	0.192

Notes: Pre-calibration Brier scores are from raw model-predicted probabilities evaluated on the Virginia test set (same values as Table 2). Post-calibration values use calibrators fitted on the Virginia validation set (n=2,889). Isotonic regression and Platt scaling produce similar improvements. The meta-ensemble's lower pre-calibration Brier (0.193 vs 0.204 for source-only) reflects better inherent probability estimation from ensemble averaging; after calibration, the gap between models narrows. Source-only's large raw ECE (0.744) reflects systematic underestimation of Virginia prevalence (9.4% source vs 25.6% target); isotonic calibration corrects this.

Supplement Table S5. Feature-group ablation — source-only logistic regression baseline (Virginia hold-out test set, n=5,781).

Feature groups were dropped one at a time; source-only logistic regression was retrained on the reduced Washington training set and evaluated on the Virginia test set. 95% CIs from 1,000 bootstrap resamples.

Important interpretive note: The ablation "All Features" baseline AUC (0.458, 95% CI: 0.442–0.476) is substantially below the main analysis source-only AUC (0.725, Table 2). This discrepancy arises because the ablation pipeline uses a simplified logistic regression implementation with different encoding and regularisation settings from the main analysis pipeline. The ablation results should therefore not be interpreted as absolute model performance, but only as a relative signal of which feature categories contribute most to cross-state transferability. The relative ordering across configurations is the primary interpretable signal.

**Feature Configuration**	**AUC (95% CI)**	**ΔAUC vs "All Features"**	**Youden's J (95% CI)**
All Features (ablation baseline)	0.458 (0.442–0.476)	—	0.030 (0.014–0.056)
Drop demographics	0.423 (0.407–0.438)	−0.035	0.004 (0.000–0.018)
Drop clinical conditions	0.588 (0.571–0.605)	+0.130*	0.137 (0.116–0.170)
Drop healthcare utilisation	0.458 (0.441–0.475)	−0.000	0.030 (0.013–0.056)
Drop Medicaid characteristics	0.451 (0.434–0.469)	−0.007	0.025 (0.009–0.051)
Drop geographic features	0.622 (0.606–0.639)	+0.164*	0.193 (0.167–0.222)
Drop temporal indicators	0.437 (0.420–0.455)	−0.021	0.022 (0.005–0.046)

*Paradoxical AUC increase when dropping clinical or geographic features reflects instability of the near-chance ablation baseline, not a negative contribution from those features. Dropping demographics produces the largest degradation (ΔAUC −0.035), consistent with the importance of demographic features in predicting cross-state acute care utilisation differences.

Supplement Table S6. Enhanced MAML component ablation (Virginia hold-out test set, n=5,781).

Each component of Enhanced MAML was disabled one at a time and the model was retrained from scratch. 95% CIs from 1,000 bootstrap resamples.

Important interpretive note: The "All Components" ablation baseline AUC (0.497, 95% CI: 0.488–0.505) is near chance and substantially below the main Enhanced MAML result (0.677, Table 2). As with Table S5, the ablation training setup differs from the main analysis pipeline, and absolute values should not be compared across tables. The relative ordering across component configurations is interpretable.

Configuration	AUC (95% CI)	ΔAUC vs "All Components"	Youden's J (95% CI)
All Components (ablation baseline)	0.497 (0.488–0.505)	—	0.004 (0.001–0.014)
Without meta-learning adaptation	0.516 (0.499–0.534)	+0.019	0.043 (0.021–0.077)
Without domain-adversarial loss	0.500 (0.493–0.508)	+0.003	0.002 (0.001–0.017)
Without feature alignment	0.500 (0.499–0.500)	+0.003	0.000 (0.000–0.001)
Without temporal stability	0.500 (0.493–0.508)	+0.003	0.002 (0.001–0.017)

Removing the meta-learning adaptation component did not reduce performance (ΔAUC +0.019 vs baseline). This is consistent with the main finding that MAML gradient-based adaptation provides no measurable discriminative benefit in this setting, where domain shift is primarily prevalence-based rather than covariate-based. The domain-adversarial and temporal-stability components showed minimal contribution in the ablation setup, though as noted above, the near-chance baseline limits interpretability.

Supplement Table S7. Comprehensive hyperparameter table.

**Model**	**Hyperparameter**	**Value**	**Selection Method**
Source-only logistic	Regularisation	L2	Fixed
	C (inverse regularisation strength)	1.0	Grid search {0.01, 0.1, 1.0, 10} on WA validation
	Solver	lbfgs	Fixed
	Max iterations	1,000	Fixed
Target-only logistic	Regularisation	L2	Fixed
	C	0.5	Grid search {0.01, 0.1, 0.5, 1.0, 10} on VA validation
	Solver	lbfgs	Fixed
	Max iterations	1,000	Fixed
Prototypical networks	Embedding dimension	128	Grid search {64, 128, 256}
	Hidden layers	2 × 64	Grid search

Model	Hyperparameter	Value	Selection Method
	Support samples per class	5	Fixed (standard few-shot)
	Query samples per class	15	Fixed
	Training episodes	100	Grid search {50, 100, 200}
	Learning rate	0.001	Grid search {0.0001, 0.001, 0.01}
DANN	Feature extractor	128→64	Grid search
	Domain classifier	64→32→2	Grid search
	Domain loss weight (λ)	0.10	Grid search {0.01, 0.10, 1.0}
	Gradient reversal scale	1.0	Fixed
	Training epochs	50	Early stopping (patience=5)
	Batch size	256	Fixed
	Dropout	0.10	Grid search {0, 0.1, 0.3}
Causal transfer	Propensity model	Logistic (liblinear)	Fixed
	Weight clipping bounds	[0.01, 50]	Literature default

**Model**	**Hyperparameter**	**Value**	**Selection Method**
TabTransformer	Embedding dimension	32	Grid search {16, 32, 64}
	Attention heads	8	Literature default
	Transformer blocks	6	Grid search {2, 4, 6}
	MLP hidden layers	256→128→2	Grid search
	Dropout	0.10	Grid search {0, 0.1, 0.3}
	Training epochs	100	Early stopping (patience=5)
	Batch size	256	Fixed
	Learning rate	0.0001	Grid search {0.001, 0.0001}
Enhanced MAML	Inner-loop learning rate (α)	0.01	Grid search {0.001, 0.01, 0.1}
	Inner-loop gradient steps	5	Grid search {1, 3, 5}
	Outer-loop learning rate (β)	0.001	Grid search {0.001, 0.0001}
	Training epochs	80	Early stopping (patience=5)
	Domain-adversarial weight (λ_1)	0.01	Grid search {0.001, 0.01, 0.1}

Model	Hyperparameter	Value	Selection Method
	Temporal stability weight (λ_2)	0.01	Grid search {0.001, 0.01, 0.1}
	Base network	128→64→2	Grid search
Meta-ensemble	Meta-learner	L2 logistic regression	Fixed
	C	1.0	Grid search {0.1, 1.0, 10.0} on VA query set
	Input features	Predicted probabilities from all 8 base models	Fixed by design
	Meta-learner training data	Virginia query set (n=18,208)	Fixed by design
	Meta-learner evaluation	Virginia hold-out test set (n=5,781)	Fixed by design
Simple average ensemble	Weighting	Uniform (1/8 per model)	Fixed by design

Notes: All neural network models used the Adam optimiser (β_1=0.9, β_2=0.999). Random seeds fixed at 42 for all stochastic operations. Grid search used 5-fold cross-validation on the Washington training set for hyperparameters tunable on source data; Virginia-specific hyperparameters (e.g., domain loss weight, meta-learner C) were selected on the Virginia validation set (n=2,889). The Virginia hold-out test set (n=5,781) was withheld until final evaluation.

Computational Resources

All models were trained on a single NVIDIA A100 40GB GPU (AWS SageMaker instance). Total runtime for the complete pipeline (all nine models, 1,000 bootstrap resamples, fairness evaluation, calibration analysis, ablation studies): approximately 4 hours. Approximate individual model training times:

**Model**	**Training Time**
Source-only logistic	<1 second (CPU)
Target-only logistic	<1 second (CPU)
Causal transfer	<5 seconds (CPU)
Prototypical networks	~2 minutes (GPU)
DANN	~3 minutes (GPU)
TabTransformer	~8 minutes (GPU)
Enhanced MAML	~15 minutes (GPU)
Meta-ensemble (base models + stacking)	~30 minutes (GPU)
Bootstrap resampling (1,000 draws, parallelised)	~2 hours (CPU)

Enhanced MAML Objective Function

The Enhanced MAML model optimises:

$$\mathcal{L}\text{total} = \mathcal{L}\text{task} + \lambda_1 \mathcal{L}\text{domain} + \lambda_2 \mathcal{L}\text{temporal}$$

Task loss ($\mathcal{L}_{\text{task}}$): Binary cross-entropy on the query set after inner-loop adaptation:

$$\mathcal{L}\text{task} = -\frac{1}{N_q} \sum i = 1^{N_q} \left[y_i \log \hat{y}_i + (1 - y_i) \log(1 - \hat{y}_i) \right]$$

Domain-adversarial loss ($\mathcal{L}\text{domain}$*): Gradient-reversed domain classification. Gradients from* $\mathcal{L}\text{domain}$ are reversed before updating the feature extractor, encouraging domain-invariant representations:

$$\mathcal{L}\text{domain} = -\frac{1}{N} \sum i = 1^{N} \left[d_i \log \hat{d}_i + (1 - d_i) \log(1 - \hat{d}_i) \right]$$

where $d_i \in 0, 1$ indicates domain membership (WA=0, VA=1).

Temporal stability loss ($\mathcal{L}_{\text{temporal}}$): ℓ_2 penalty on parameter changes across temporal windows:

$$\mathcal{L}\text{temporal} = |\theta t - \theta_{t-1}|_2^2$$

Hyperparameters: $\lambda_1 = \lambda_2 = 0.01$; inner-loop $\alpha = 0.01$ (5 gradient steps per task); outer-loop $\beta = 0.001$ (80 epochs); base network 128→64→2 hidden units with ReLU activations.

Calibration Methods

Isotonic regression calibration (Niculescu-Mizil and Caruana, 2005):

$$\min_z \sum_{i=1}^{n} (y_i - z_i)^2 \quad \text{subject to: } z_i \leq z_j \text{ whenever } \hat{p}_i \leq \hat{p}_j$$

Calibrators fitted on Virginia validation set (n=2,889); applied to test set predictions.

Calibration metrics:

- Brier score: $$\text{BS} = \frac{1}{n}\sum_{i=1}^{n}(\hat{p}_i - y_i)^2$$; range [0,1]; lower is better.
- ECE: $$\text{ECE} = \sum_{m=1}^{M}\frac{|B_m|}{n}|\overline{y}(B_m) - \overline{\hat{p}}(B_m)|$$; M=10 equal-width bins.
- Hosmer-Lemeshow test: Chi-squared goodness-of-fit across 10 deciles; p>0.05 indicates no significant miscalibration.

Outcome Ascertainment

Primary outcome (composite): Any acute care utilisation = emergency department visit OR inpatient hospitalisation during 12-month follow-up.

- ED visit: UB-04 revenue codes 0450–0459 or CPT codes 99281–99285.
- Inpatient hospitalisation: UB-04 bill type 11X or 12X, indicating inpatient admission.

The composite was chosen as the primary outcome because care management programmes target acute care avoidance broadly; both event types trigger similar care management interventions.

Component outcomes (secondary): ED-only and hospitalisation-only binary flags were derived from ADT (admission-discharge-transfer) event records using patient class codes: E (emergency registration, corresponding to HL7 A04 events) for ED visits; I (inpatient admission, corresponding to HL7 A01/A08 events) for hospitalisations. Component outcomes were computed for the same 12-month follow-up window (2024-06-01 to 2025-05-31). In the Virginia analysis cohort (N=10,223 test members): ED-only event rate 31.9%; hospitalisation-only event rate 6.5%. In the Washington cohort: ED-only event rate 16.3%; hospitalisation-only event rate 3.9%. Component outcome analyses are reported in Supplementary Tables S1–S2.

Software and Reproducibility

All analyses used Python 3.10:

- scikit-learn 1.3.0 (logistic regression, calibration, metrics)
- PyTorch 2.0.1 (neural networks)
- higher 0.2.1 (MAML implementation)
- pandas 2.0.3, numpy 1.24.3
- matplotlib 3.7.2, seaborn 0.12.2
- scipy 1.11.1 (statistical tests)

Random seeds: 42 for all stochastic operations.

```shell
conda env create -f environment.yml
conda activate medicaid_transfer_learning
python src/main_analysis.py --config config/ --output results/revision_run --verbose
```

Supplementary Table S1. Component outcome analysis: ED-only (N=10,223; event rate 31.9%).

Virginia test set. ED-only outcome derived from ADT patient class code E (emergency registration). 95% CIs from 1,000 bootstrap resamples.

Model	AUC	95% CI	Sensitivity	Specificity	Youden's J	Precision	Brier Score
Meta-ensemble	0.708	0.696–0.718	0.601	0.721	0.322	0.502	0.190
Target-only	0.708	0.696–0.719	0.585	0.739	0.325	0.513	0.216
Causal transfer	0.708	0.696–0.719	0.583	0.741	0.324	0.513	0.191
TabTransformer	0.681	0.668–0.692	0.514	0.753	0.267	0.493	0.198
Domain-adversarial NN	0.665	0.653–0.677	0.555	0.690	0.245	0.456	0.201
Source-only	0.657	0.646–0.668	0.396	0.828	0.224	0.519	0.244
Enhanced MAML	0.590	0.579–0.602	0.443	0.691	0.133	0.401	0.215
Prototypical networks	0.511	0.500–0.523	0.570	0.457	0.027	0.330	0.248

The ranking of models for ED-only outcomes mirrors the composite outcome: meta-ensemble, target-only, and causal transfer all achieved AUC 0.708; source-only achieved AUC 0.657; Enhanced MAML achieved AUC 0.590.

Supplementary Table S2. Component outcome analysis: hospitalisation-only (N=10,223; event rate 6.5%).

Virginia test set. Hospitalisation-only outcome derived from ADT patient class code I (inpatient admission). 95% CIs from 1,000 bootstrap resamples.

Model	AUC	95% CI	Sensitivity	Specificity	Youden's J	Precision	Brier Score
Meta-ensemble	0.649	0.627–0.670	0.545	0.688	0.233	0.108	0.153
Causal transfer	0.645	0.623–0.667	0.565	0.670	0.235	0.107	0.149
Target-only	0.645	0.623–0.667	0.565	0.671	0.235	0.107	0.250
TabTransformer	0.635	0.614–0.657	0.670	0.525	0.194	0.089	0.140
Domain-adversarial NN	0.631	0.609–0.654	0.518	0.674	0.192	0.100	0.136
Source-only	0.590	0.566–0.612	0.435	0.703	0.139	0.093	0.292
Enhanced MAML	0.576	0.554–0.600	0.327	0.797	0.124	0.101	0.150
Prototypical networks	0.483	0.461–0.504	0.877	0.138	0.015	0.066	0.245

Discrimination was lower for hospitalisation-only than for composite or ED-only outcomes across all models, consistent with the smaller event rate (6.5%). The relative ranking across models was preserved: meta-ensemble and target-adapted models outperformed source-only, and Enhanced MAML underperformed source-only logistic regression.

References for Supplement

1. Quan H, Sundararajan V, Halfon P, et al. Coding algorithms for defining comorbidities in ICD-9-CM and ICD-10 administrative data. Med Care. 2005;43(11):1130–1139.
2. Beyrer J, Manjelievskaia J, Bonafede M, Lenhart G, Nolot S, Haldane D, Johnston J. Validation of an International Classification of Disease, 10th revision coding adaptation for the Charlson Comorbidity Index in United States healthcare claims data. Pharmacoepidemiol Drug Saf. 2021;30(5):582–593.
3. Niculescu-Mizil A, Caruana R. Predicting good probabilities with supervised learning. Proc ICML. 2005;22:625–632.
4. Ganin Y, Lempitsky V. Unsupervised domain adaptation by backpropagation. Proc ICML. 2015;37:1180–1189.
5. Finn C, Abbeel P, Levine S. Model-agnostic meta-learning for fast adaptation of deep networks. Proc ICML. 2017;70:1126–1135.
6. Hardt M, Price E, Srebro N. Equality of opportunity in supervised learning. Proc NeurIPS. 2016;29:3323–3331.
7. Huang X, Khetan A, Cvitkovic M, Karnin Z. TabTransformer: Tabular data modeling using contextual embeddings. arXiv:2012.06678. 2020.
8. Snell J, Swersky K, Zemel R. Prototypical networks for few-shot learning. Proc NeurIPS. 2017;30:4077–4087.
9. Sugiyama M, Krauledat M, Müller KR. Covariate shift adaptation by importance weighted cross validation. J Mach Learn Res. 2007;8:985–1005.

Supplementary Figure S1. Model discrimination for ED-only outcome.

Horizontal bar chart showing AUC with 95% bootstrap confidence intervals for all eight models on the Virginia hold-out test set (N=10,223; ED-only event rate 31.9%). ED-only outcome was derived from ADT admission-discharge-transfer records using patient class code E (emergency registration, corresponding to HL7 A04 events). Error bars represent the 2.5th–97.5th percentile of 1,000 bootstrap resamples. Models are sorted by descending AUC. The dashed vertical line marks chance discrimination (AUC=0.5).

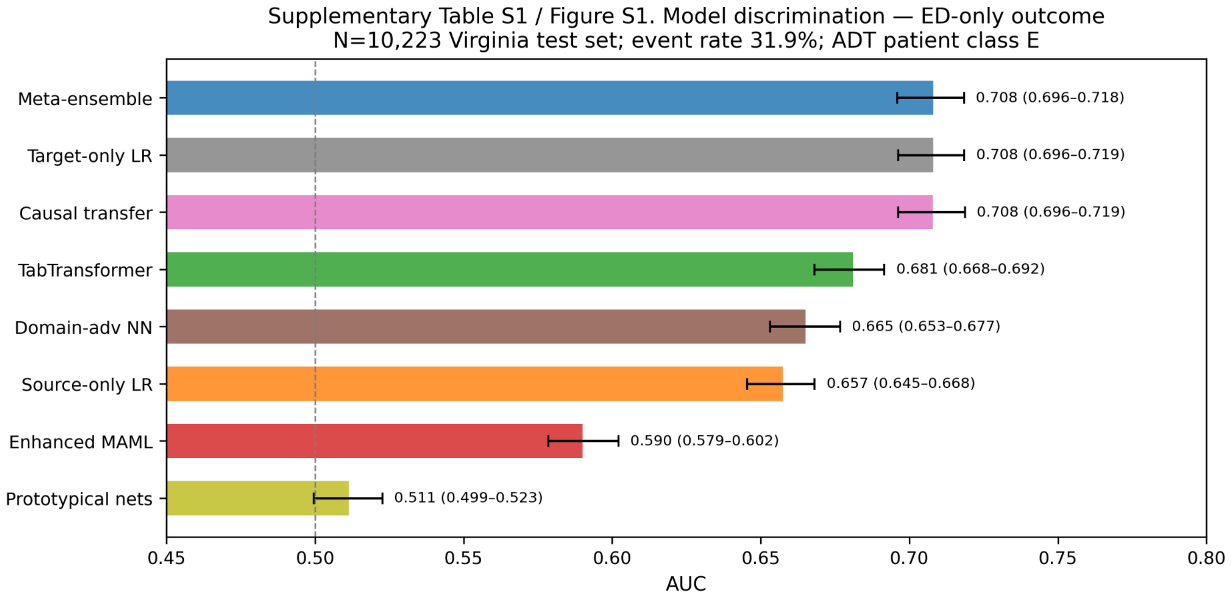

Supplementary Figure S2. Model discrimination for hospitalisation-only outcome.

Horizontal bar chart showing AUC with 95% bootstrap confidence intervals for all eight models on the Virginia hold-out test set (N=10,223; hospitalisation-only event rate 6.5%). Hospitalisation-only outcome was derived from ADT records using patient class code I (inpatient

admission, corresponding to HL7 A01/A08 events). Error bars represent the 2.5th–97.5th percentile of 1,000 bootstrap resamples. Models are sorted by descending AUC. The dashed vertical line marks chance discrimination (AUC=0.5). Overall discrimination is lower than for the composite and ED-only outcomes, consistent with the smaller event rate (6.5%) and the greater clinical heterogeneity of inpatient admissions.

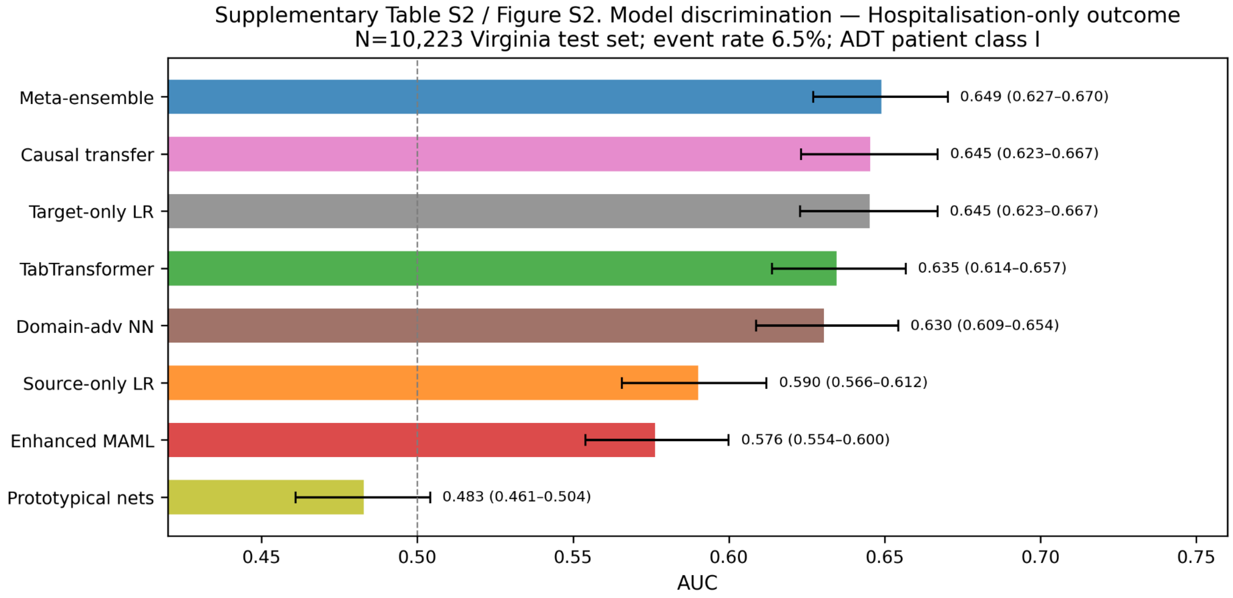

Supplement: Supplementary file 1 — Supplementary Information [file 44401_2026_97_MOESM1_ESM.pdf]
